# Biomarkers in Atopic Dermatitis in Children: A Comprehensive Review

**DOI:** 10.3390/life15030375

**Published:** 2025-02-27

**Authors:** Cristiana Indolfi, Carolina Grella, Angela Klain, Giulio Dinardo, Simone Colosimo, Dario Piatto, Claudia Nespoli, Alessandra Perrotta, Michele Miraglia del Giudice

**Affiliations:** Department of Woman, Child and General and Specialized Surgery, University of Campania ‘Luigi Vanvitelli’, 80138 Naples, Italy; cristianaind@hotmail.com (C.I.); caro.grella94@gmail.com (C.G.); simone.colosimo@studenti.unicampania.it (S.C.); dariopiatto3@yahoo.com (D.P.); nespoliclaudia10@gmail.com (C.N.); alessandraperrotta96@gmail.com (A.P.); michele.miragliadelgiudice@unicampania.it (M.M.d.G.)

**Keywords:** atopic dermatitis, biomarkers, pediatric allergy, atopic march, skin barrier dysfunction, cytokines, microbiome, personalized medicine

## Abstract

Atopic dermatitis (AD) is a chronic inflammatory skin disorder with significant implications for patient quality of life and a well-documented association with the atopic march. Recent advancements in biomarker research have unveiled critical insights into AD pathogenesis, diagnosis, prognosis, and therapeutic monitoring. This comprehensive review evaluates the utility of emerging biomarkers, including cytokines, chemokines, genetic markers, and microbiome-related components, in understanding the disease mechanisms and stratifying patient care. The role of minimally invasive diagnostic techniques, such as tape stripping and RNA monitoring, is highlighted, offering innovative approaches to pediatric populations. Furthermore, this review explores the biomarkers that predict disease progression, therapeutic response, and comorbidities, including food allergies and asthma. Personalized treatment strategies based on endotype-specific biomarkers are discussed as a future direction for improving clinical outcomes. Despite promising findings, the integration of biomarkers into routine practice necessitates further validation through large-scale studies. This work underscores the transformative potential of biomarker-driven approaches in enhancing the management of AD in children and its associated conditions.

## 1. Introduction

### 1.1. Atopic Dermatitis and the Atopic March

Atopic dermatitis (AD) is a chronic, recurrent inflammatory disease that affects the skin, characterized by inflammation, eczematous lesions, and itching that does not respond to antihistamines. In 45% of cases, onset occurs before 6 months of age, affecting up to 25% of children and 10% of adults [1,2,3,4]. AD is often the first sign of the progressive sequence of allergic diseases known as the “atopic march.” This concept, introduced 20 years ago, describes the sequential development of atopic manifestations, with AD predisposing individuals to asthma and allergic rhinitis [5]. Epidemiological studies and murine models have shown a connection between AD and subsequent allergic airway inflammation, suggesting that skin barrier dysfunction and inflammation may promote allergen sensitization. The original definition of the “atopic march” has been expanded to include other conditions, such as food allergy (FA), asthma, rhinitis, and, to a lesser extent, eosinophilic esophagitis. AD is considered a “gateway” for these comorbidities, with genetic factors, environmental influences, and microbiome alterations contributing to the risk. However, not all children with AD develop other atopic diseases: approximately 50% do not follow this progression [6].

The atopic march serves as a paradigm for understanding the natural progression of Th2 lymphocyte-mediated allergic diseases. Despite the hypothesis of a standardized progression, with AD as the first step, clinical practice shows significant variability in the pathways and sequence of allergic diseases. These pathways, influenced by environmental, genetic (such as filaggrin (FLG) gene mutations), and psychosocial factors, share Th2 responses characterized by IgE production, granulocyte activation, edema, and mucus production [7]. Preventing AD could, therefore, reduce the risk of developing other allergies. Protective environmental factors include vaginal delivery and breastfeeding, while early exposure to antibiotics, irritants, and skin dysbiosis (e.g., colonization by *Staphylococcus aureus*) increases the risk [8,9]. Recent studies highlight the role of IL-13 in skin barrier dysfunction, making it a key mediator of the Th2 response [10]. The epithelial barrier theory links exposure to modern toxins (detergents, pollution, and microplastics) to epithelial damage, microbial dysbiosis, and inflammation, fostering a type 2 immune response involving eosinophils, mast cells, and other immune cells. These damages, associated with environmental changes, contribute not only to allergic and autoimmune diseases but also to a range of systemic conditions. The progression from AD to other allergies, including asthma, is not universal but reflects complex and diverse pathways in which genetic, environmental, and temporal factors play integrated roles [7].

### 1.2. Pathogenesis and Immune Mechanisms

The pathogenesis of AD is multifactorial, involving the interaction between epidermal barrier dysfunction, genetic predisposition, and environmental factors, with an immune response skewed toward type 2 (T2) and type 17 (T17) T-cell responses [11]. The mutations most strongly associated with AD are those in the FLG gene, which weakens the skin barrier, increasing susceptibility to the disease, exacerbated by environmental factors such as pollution and excessive soap use [12,13]. Other mutations involve skin barrier proteins such as SPINK and claudins [12,14]. The immune response mediated by T lymphocytes and changes in cytokine patterns are crucial in the pathogenesis of AD. The compromised skin barrier facilitates exposure to environmental allergens and activates epidermal dendritic cells and keratinocytes, promoting the T2 response and the production of specific IgE. In chronic cases of atopic dermatitis, there is a predominance of TH1 and TH17 immune responses. These T-cell subtypes release proinflammatory cytokines such as interferon-gamma (IFN-γ), tumor necrosis factor-alpha (TNF-α), and interleukin-17 (IL-17), which intensify inflammation and impair the skin barrier, worsening disease symptoms [11,15]. To counteract this inflammation, dupilumab, an IL-4 receptor antagonist, simultaneously blocks the signals mediated by IL-4 and IL-13, two key cytokines in the type 2 immune response, thereby reducing inflammation and improving skin barrier function. JAK inhibitors (such as abrocitinib, baricitinib, and upadacitinib) act by modulating the JAK/STAT signaling pathway, which is critical for transmitting signals from numerous cytokines involved in chronic inflammation. By blocking this pathway, JAK inhibitors significantly reduce inflammatory cytokine levels, alleviating symptoms, reducing itching, and improving the quality of life in patients with moderate-to-severe atopic dermatitis [16,17]. An important role in the pathogenesis of AD is also played by the alteration of the skin microbiome, which affects the periodicity of the disease, as it is characterized by periods of the worsening and improvement of skin lesions. In AD, the composition of the skin microbiome changes during flare-ups, with a reduced microbial diversity and an increase in *Staphylococcus aureus* (*S. aureus*), which plays a key role in exacerbating skin inflammation. This bacterium is more prevalent on lesional skin, while *S. epidermidis* predominates during the improvement phase. Non-lesional skin of patients with AD shows an altered microbial composition compared to healthy individuals, contributing to the susceptibility to flare-ups. Moreover, *S. aureus* is associated with skin barrier dysfunction and inflammatory processes, with more virulent strains observed in patients with more severe disease. Colonization by *S. aureus* is linked to genetic and immune factors, including defects in skin barrier genes such as FLG, which promote bacterial adhesion. The interaction between viruses and bacteria, such as *S. aureus* and the herpes simplex virus, increases morbidity in patients with AD. The fungal composition in AD is also altered, with a reduction in *Malassezia* and an increase in fungi such as *Aspergillus*. Some studies suggest that *Malassezia globosa* may interfere with the growth of *S. aureus*, but the impact on the skin barrier is still unclear. Other commensal microorganisms, such as *S. epidermidis*, play a role in defending against pathogens by stimulating the production of antimicrobial peptides [18].

### 1.3. Clinical Manifestations, Diagnosis, and Treatment

AD lesions undergo significant changes in morphology and distribution as patients age. In infants, infantile seborrheic dermatitis is one of the most common eruptions, primarily affecting the face and scalp. These children also present dry skin, with a gooseflesh-like appearance, and eczema is localized mainly in the antecubital and popliteal fossae. These manifestations are typical of AD in young children, an age at which lesions are mostly limited to these areas. As patients age, the lesions become more complex. In older children and adolescents, the skin lesions tend to become drier and more lichenified, with thickening and a rough texture of the skin. Furthermore, adult patients may develop characteristic signs such as “red face”, presenting as persistent dark reddish erythema on the face, and “dirty neck”, with reticulated and poikilodermatous lesions on the neck. These clinical changes between pediatric and adult patients are likely related to differential activation and infiltration of T-cell subsets in the skin, which changes with age. Studies on the evolution of pathological T-cell subsets in AD, particularly in peripheral blood lymphocytes, have revealed significant differences between age groups [19,20].

In infants with AD, a low frequency of cutaneous lymphocyte-associated antigen (CLA)+ Th1 cells is observed, while the frequency of CLA+ Th2 cells is increased. This suggests that Th2 immune response predominates during the early years of life. As patients age, particularly in adolescents and adults, the immune response evolves, with an increase in IL-22 frequencies, a cytokine involved in the pathogenesis of AD, and a progressive involvement of the TH22, TH17, and TH1 pathways. These pathways are associated with a weakened epidermal barrier, characteristic of AD in adults. However, in pediatric patients, Th1 activation is less pronounced, and defects in the epidermal lipid metabolism contribute more significantly to skin barrier damage. This explains the difference in clinical manifestations between adults and children, with children tending to present more limited and less lichenified lesions compared to adults [19]. Individuals with AD are genetically predisposed to develop allergic diseases such as allergic rhinitis, hay fever, asthma, atopic dermatitis, and food allergies, with a strong family component. Diagnosis is based on a thorough history, physical examination, and diagnostic tests, such as measuring immunoglobulin E (IgE) levels and, in the case of suspected food allergies, oral provocation tests [21]. The severity of AD can be assessed using systems such as the Scoring of Atopic Dermatitis (SCORAD) and the Eczema Area Severity Index, but these subjective methods are affected by inter- and intra-observer variability. To overcome these limitations, circulating biomarkers, measurable through a simple venous blood draw, can represent a more objective and reliable alternative [22].

Given the complexity of atopic dermatitis and its connection to other allergic diseases, our study aims to investigate the specific biomarkers involved in the progression of AD and its response to treatment. By identifying the predictive factors associated with clinical outcomes, we seek to contribute to a more precise stratification of patients and the development of targeted therapeutic strategies, ultimately enhancing disease management.

## 2. Materials and Methods

**Figure 1:**
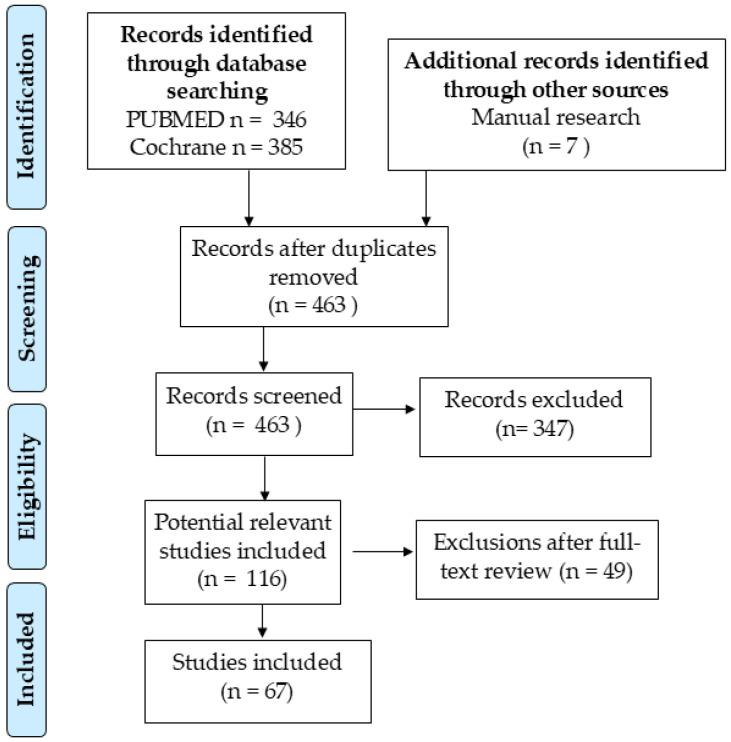
Search algorithm process.

This review was conducted as a narrative synthesis of the available literature to investigate the role of biomarkers in pediatric AD. The literature search was performed using the PubMed and Scopus databases between October 2024 and January 2025. The studies considered for inclusion were those published in English from 2014 to January 2025. Keywords employed in the search included “Atopic Dermatitis”, “Children”, “Biomarkers”, “Pediatric Allergy”, “Atopic March”, “Skin Barrier Dysfunction”, and “Non-Invasive Diagnostics.” Priority was given to clinical trials, systematic reviews, and meta-analyses relevant to the pathogenesis, diagnosis, prognosis, and treatment of AD. Three independent reviewers (G.D., C.G., and A.K.) evaluated the eligibility of the studies, with a fourth reviewer (C.I.) consulted in cases of disagreement. Consensus was achieved between at least three reviewers regarding the specific data extracted, focusing on biomarker identification, diagnostic tools, and therapeutic implications (Figure 1).

## 3. Results

Biomarkers have emerged as essential tools in unraveling the intricate mechanisms underlying AD, offering insights into the disease pathogenesis, severity, progression, and therapeutic responses. By identifying distinct molecular signatures and immune pathways, biomarkers facilitate a more precise stratification of patients, enabling personalized treatment approaches, early intervention strategies, and the development of targeted therapies, including biologics and small molecules. In our research, we categorized the biomarkers into the following five key groups: diagnostic, prognostic, severity assessment, predictive, and comorbidity biomarkers for AD (Table 1) [23].

### 3.1. Diagnostic Biomarkers

Recent advancements have identified several biomarkers that enhance the differential diagnosis of AD, aiding in its distinction from conditions like psoriasis and other eczematous disorders.

#### 3.1.1. Carbonic Anhydrase II (CA II) and Neuron-Specific Nel-like Protein 2 (NELL2)

Among these, the carbonic anhydrase II (CA II) gene is markedly upregulated in eczematous lesions, serving as a useful marker to rule out psoriasis, which lacks such expression [24,25]. Similarly, neuron-specific Nel-like protein 2 (NELL2), prominently expressed in the epidermis of AD patients, has been linked to the pruritus characteristic of the disease, offering a potential target for diagnostic and therapeutic strategies [25].

#### 3.1.2. Urinary Lipid Profile Analysis

Innovative sampling techniques, including urinary lipid profile analysis, have also emerged as valuable tools. Elevated levels of prostaglandins (e.g., PGF2α, PGE2, and PGD2) and arachidonic acid metabolites in the urine of AD patients reflect systemic inflammation and its broader metabolic implications [26].

#### 3.1.3. NOS2 and CCL27 (CTACK)

Additionally, markers like NOS2 and CCL27 (CTACK) have shown significant utility in distinguishing AD from psoriasis. While these biomarkers are upregulated in psoriasis, they are typically downregulated in AD, improving the diagnostic precision for psoriasiform dermatitis [27,28].

#### 3.1.4. IL-36γ and Human Beta-Defensin 2 (hBD-2)

Other markers further refine this differentiation; for instance, IL-36γ, which is significantly elevated in psoriasis lesions but less specific to AD, and human beta-defensin 2 (hBD-2), which correlates strongly with psoriasis severity but is minimally expressed in AD [27,28,29,30,31].

#### 3.1.5. Matrix Metalloproteinases (MMP-8 e MMP-9)

Matrix metalloproteinases, specifically MMP-8 and MMP-9, have also been identified as active in AD lesions, reflecting tissue remodeling and inflammation. Their levels correlate with disease activity, providing additional diagnostic and prognostic insights [32].

### 3.2. Prognostic and Screening Biomarkers

While diagnostic biomarkers primarily focus on distinguishing AD from other dermatological conditions, prognostic biomarkers provide insights into disease progression, risk factors, and potential complications. These categories, although distinct, are interconnected, as early and accurate diagnosis often informs the prediction of disease outcomes and guides timely interventions. The following section delves into prognostic biomarkers, highlighting their role in predicting AD persistence, comorbidities, and the atopic march [3,4,7]. Research into prognostic biomarkers has provided critical insights into disease progression, risk prediction, and early intervention strategies [2]. These biomarkers also shed light on the systemic mechanisms driving the atopic march from the skin to other organs, including the gastrointestinal and respiratory systems [33,34].

#### 3.2.1. Filaggrin Mutations

FLG mutations are among the most well-established genetic risk factors for AD [35]. These mutations compromise the skin barrier, leading to severe early-onset AD, and increasing susceptibility to food allergies and the atopic march [36,37,38,39,40].

#### 3.2.2. Natural Moisturizing Factor (NMF) and Transepidermal Water Loss (TEWL)

Reduced levels of natural moisturizing factor (NMF), often linked to FLG mutations, further reflect impaired skin integrity and heighten the risk of AD development, particularly in neonates [40,41,42,43]. Non-invasive measures such as transepidermal water loss (TEWL) provide additional predictive value, with high TEWL in newborns signaling skin barrier dysfunction and increased AD risk within the first year of life [41,42].

#### 3.2.3. Vascular Endothelial Growth Factor (VEGF) and Indoleamine 2,3-Dioxygenase-1 (IDO1)

Emerging biomarkers like vascular endothelial growth factor (VEGF) and indoleamine 2,3-dioxygenase-1 (IDO1) have revealed new dimensions of disease monitoring. Low VEGF levels are linked to persistent AD, while IDO1 offers potential insights into complications such as eczema herpeticum [44].

#### 3.2.4. MicroRNAs (miR-155, miR-203, and miR-483-5p) and Succinate mtDNA

MicroRNAs (miRNAs) have also garnered attention as novel biomarkers. Elevated miR-155 levels strongly correlate with AD severity in children [45], while miR-203 and miR-483-5p in serum and urine provide promising avenues for the non-invasive monitoring of inflammation and disease progression [46,47]. Additionally, lipid and cytokine alterations, such as reduced ceramide levels in the stratum corneum, highlight the critical role of skin barrier dysfunction in initiating immune activation [48,49]. In addition, succinate and mitochondrial DNA (mtDNA) have been identified as key mediators in the progression of AD to food allergies and intestinal inflammation [50]. Tissue damage in AD results in the release of these molecules, which act as danger signals that drive systemic inflammatory responses [50]. Succinate, released from damaged tissues, promotes type 2 inflammation in the gut through the succinate–tuft cell–ILC2 (innate lymphoid cell) axis. Succinate activates tuft cells in the gut epithelium, leading to the expansion of ILC2s and the release of inflammatory cytokines such as IL-25, which amplifies the type 2 immune response. Elevated succinate levels were observed in both a mouse model of AD and in clinical serum samples from children with AD, confirming its role in the atopic march [50]. Mitochondrial DNA, released after tissue damage, activates the STING (stimulator of interferon genes) pathway, further amplifying inflammation. This pathway contributes to the progression of inflammation from the skin to the gut, as observed in a mouse model of mechanical skin injury and allergen sensitization. Clinical data corroborated these findings, with children with AD displaying higher serum levels of mtDNA compared to non-AD allergic children [50]. The atopic march is also associated with alterations in gut microbiota. Inflammatory processes reshape the gut microbiota, increasing the abundance of succinate-producing bacteria. These changes further enhance systemic inflammation by driving higher succinate production and absorption in the gut. This interplay between gut microbiota and systemic inflammation highlights the importance of microbiota–immune system interactions in the progression of AD to other allergic conditions [51,52]. The identification of succinate and mtDNA as biomarkers offers potential strategies for the early risk assessment and targeted prevention of the atopic march. Both molecules serve as critical mediators of the systemic inflammation that drives disease progression, making them promising targets for therapeutic intervention. However, further large-scale studies are needed to validate these findings and integrate them into clinical practice.

### 3.3. Biomarkers for Severity Assessment in Atopic Dermatitis

Recent studies have demonstrated that certain biomarkers play a pivotal role in understanding the pathogenesis, severity, and treatment responses in AD [53,54].

#### 3.3.1. Th2/Th22 Immune Mediators

Among these, the most critical are cytokines associated with the Th2 and Th22 immune pathways, such as IL-13 and IL-22, as well as key chemokines, including thymus and activation-regulated chemokine/C-C motif ligand 17 (TARC/CCL17), eotaxin-3/CCL26, cutaneous T-cell-attracting chemokine (CTACK/CCL27), pulmonary and activation-regulated chemokine (PARC/CCL18), and macrophage-derived chemokine (MDC/CCL22), which highlight the immune dysregulation central to AD pathogenesis [2,55]. A key advancement in biomarker research is the use of tape stripping, a minimally invasive method that has enabled the identification of transcriptomic signatures unique to lesional and non-lesional AD skin [56,57]. These transcriptomic insights have revealed that non-lesional skin, despite appearing clinically normal, exhibits distinct immunological activity, including the upregulation of type 2 high gene signatures, which correlates with systemic inflammation and disease severity. The findings include the significant overexpression of genes linked to Th2 and Th22 responses, such as IL-4, IL-13, IL-22, and related chemokines (TARC/CCL17, eotaxin-3/CCL26, and CTACK/CCL27) [58,59].

#### 3.3.2. IL-8 and CTACK

Moreover, biomarkers tied to pruritus, such as IL-8 and CTACK, exhibit strong associations with patient-reported outcomes like the itching intensity, emphasizing their relevance for therapeutic targeting [60,61].

#### 3.3.3. IL-1β and Thymic Stromal Lymphopoietin (TSLP)

Markers like IL-1β and thymic stromal lymphopoietin (TSLP) also show strong correlations with the SCORAD and TEWL scores, reinforcing their role in barrier impairment and disease activity [62,63].

#### 3.3.4. Th17/Th22 Cytokines, DPP-4, and Other Immune Markers

Emerging evidence suggests that Th17/Th22 cytokines (e.g., IL-17F, IL-22, and IL-26) and innate immune markers (e.g., MMP12 and S100 proteins) are crucial contributors to disease severity. Additionally, regulatory T-cell markers, such as FOXP3, reflect the dynamic immune imbalances in AD, providing further dimensions for understanding disease heterogeneity [57,64,65]. Biomarkers like DPP-4 have emerged as endotype-specific markers, with data from the ProRaD cohort linking them to clinical features such as keratosis pilaris, perleche, and eosinophilia, highlighting the systemic impact of inflammation even in milder disease (EASI < 16) [10]. Additionally, IL-13, periostin, and DPP-4 are associated with distinct phenotypes; for example, elevated IL-13 correlates with higher eosinophil counts and moderate AD severity (EASI 5.5–17), while periostin reflects Th2-driven inflammation, though its predictive value for severity needs further validation. Notably, while mild cases often fail to exhibit marked biomarker changes, severe AD demonstrates consistent upregulation across serum and lesional skin [10,55,66]. Machine learning models have further refined the association of these biomarkers with clinical phenotypes, aiding in stratifying patients for targeted treatments like IL-13 antagonists. Despite these advancements, certain biomarkers have demonstrated limited utility. For example, the “itch cytokine”, IL-31, has shown weak or inconsistent correlations with AD severity [36,67]. Toll-like receptor 2 (TLR2) polymorphisms, particularly the TLR2–16934A>T variant, have been identified as predictors of AD severity, with significant associations with SCORAD scores [68]. Similarly, markers like serum E-selectin (SELE), IL-18, and eosinophil cationic protein (ECP) are emerging as potential candidates for tracking disease activity [65,69]. Systemic inflammation markers such as serum lactate dehydrogenase (LDH) and peripheral eosinophil counts offer additional layers of insight, correlating with heightened immune activation [70]. Elevated levels of eosinophil-derived neurotoxin (EDN) and serum proteins like squamous cell carcinoma antigen 2 (SCCA2) further distinguish severe AD, with SCCA2 proving to be particularly reliable across age groups due to the minimal age-related variability [71]. These biomarkers outperform traditional measures like total serum IgE, which, while often elevated in extrinsic AD, shows only a weak or inconsistent correlation with the disease severity [2,72].

#### 3.3.5. Role of the Skin Microflora

The imbalance of skin microflora, marked by the predominance of Staphylococcus aureus and Malassezia furfur, remains a critical factor influencing the disease progression. Recent studies have suggested that high baseline levels of Staphylococcus aureus and Malassezia furfur are associated with disease severity [8], as the abundance of this pathogen exacerbates immune activation. However, methodological inconsistencies in sampling and analysis remain barriers to its broader clinical application as a biomarker [73].

### 3.4. Predictive Biomarkers

The pathophysiology of AD is multifaceted and involves various immune pathways, leading to varying therapeutic outcomes among patients [2]. As a result, identifying biomarkers that can predict how a patient will respond to a specific treatment, particularly targeted therapies, is highly valuable [36]. These predictive biomarkers could either apply broadly across all treatments (biomarkers for general disease response) or be tailored to a specific treatment (treatment-specific biomarkers), helping to identify subgroups of patients most likely to benefit from a given therapy [36]. The variations in the serum levels of these biomarkers are strongly correlated with significant treatment responses. By using qPCR, microarray, and ELISA, it is possible to determine ROC-AUC values based on the disease severity, highlighting their high predictive potential [2,36].

#### 3.4.1. Periostin and DPP-4

Elevated serum levels of periostin DPP-4 have been identified as significant biomarkers to predict favorable outcomes in patients with AD undergoing anti-IL-13 therapy (tralokinumab) [74].

#### 3.4.2. IL-22, CXCL9, CXCL2, and MDC/CCL22

Similarly, the high baseline tissue levels of IL-22 have been proposed as potential indicators of the therapeutic response to IL-22 inhibitors (fezakinumab) [75]. Additionally, CXCL9 (a Th1/interferon-associated cytokine) and CXCL2 (a Th17-associated cytokine) have been suggested as specific predictive markers for treatment with cyclosporine and dupilumab, respectively [76]. Furthermore, MDC/CCL22 has been recognized as a general disease response biomarker, independent of the therapeutic approach or targeted pathway [76]. The inflammatory response and immune profile of AD patients vary by age and ethnicity, influencing the predictive biomarkers for the treatment response [2]. While both children and adults show excessive Th2 activation, children also exhibit higher Th9 and Th17 activity, whereas adults display increased Th22 inflammation [77]. These differences suggest age-specific therapies targeting distinct cytokines [78]. Similarly, ethnic variations in cytokine expression, higher Th17/Th22 in Asians, predominant Th2/Th22 with lower Th1/Th17 in European Americans, and Th2/Th22 dominance with reduced Th1/Th17 in African Americans, may impact the treatment outcomes across groups [79,80,81].

### 3.5. Biomarkers for Comorbidities in Patients with Atopic Dermatitis

#### 3.5.1. VEGF and Indoleamine 2,3-Dioxygenase-1 (IDO1)

A low serum VEGF level has been found to predict the AD persistence in infancy [44], while the enzyme indoleamine 2,3-dioxygenase-1 (IDO1) has been proposed as a prognostic candidate biomarker for the development of eczema herpeticum and other viral complications in AD patients [82].

#### 3.5.2. KRT5, KRT14, KRT16, and FLG

In pediatric patients with AD, KRT5, KRT14, KRT16, FLG breakdown products, and AD clinical severity were predictive of concomitant food allergy [83]. FLG mutations with suppressed levels of FLG expression predispose to AD, but are also associated with other diseases, including asthma, irritant and allergic contact dermatitis, and alopecia areata [84]. High levels of IgE and dysfunctional/low levels of FLG may predispose patients with AD to food allergy as part of the atopic march [37].

#### 3.5.3. Succinate and Mitochondrial DNA (mtDNA)

Succinate and mtDNA play a crucial role in the progression of systemic inflammation [50]. Their elevated concentration in the blood of children with atopic dermatitis (compared to allergic children without AD) suggests that they could serve both as early indicators of the comorbidity risk (e.g., food allergies or asthma) and as therapeutic targets to prevent the progression of the atopic march. Specifically, succinate promotes intestinal inflammation by activating tuft cells and ILC2, amplifying the type 2 immune response [85]. Mitochondrial DNA triggers the activation of the STING pathway, contributing to inflammatory responses in multiple tissues [86]. The identification of these biomarkers therefore provides new opportunities for targeted and personalized interventions in patients with atopic dermatitis.

**Table 1:** Key biomarkers in atopic dermatitis: categories, functions, and clinical relevance.

Biomarker	Category	Function	Reference	Technique for Measuring Biomarkers in Blood
IL-13, IL-22	Serum-Specific, Severity, Predictive	Key cytokines in the Th2/Th22 pathways; correlate with disease severity and predict responses to IL-13/IL-22 inhibitors.	Thijs et al. [55], Brunner et al. [75]	ELISA, Multiplex Immunoassay (Luminex), qPCR
IL-36	Skin-Specific, Severity	Associated with neutrophilic inflammation and disease severity in AD.	Otobe et al. [30]	ELISA, qPCR
TARC/CCL17	Serum-Specific, Severity, Diagnostic	Biomarker for inflammation and treatment response; correlates with the SCORAD score and disease activity.	Mastraftsi et al. [2], Angelova-Fischer et al. [69]	ELISA, Luminex
FLG mutations	Skin-Specific, Prognostic	Associated with early-onset AD, barrier dysfunction, and progression to the atopic march.	Paternoster et al. [35], Irvine et al. [84]	PCR, NGS (Next-Generation Sequencing), Sanger Sequencing
VEGF	Serum-Specific, Prognostic	Low levels predict persistent AD in infants; involved in skin vascularization.	Lauffer et al. [44]	ELISA, Luminex, Western Blot
NELL2	Skin-Specific, Diagnostic	Linked to pruritus and overexpressed in AD epidermis, aiding in the differentiation from psoriasis.	Kamsteeg et al. [25]	ELISA, Western Blot, qPCR
MMP-8, MMP-9	Skin-Specific, Diagnostic, Severity	Reflect tissue remodeling and inflammation; correlate with AD lesion activity.	Harper et al. [32]	ELISA, Luminex, Zymography
MMP-12	Skin-Specific, Severity	Reflects tissue remodeling and correlates with AD lesion severity.	Yu et al. [65]	ELISA, Zymography
Succinate, mtDNA	Serum-Specific, Prognostic, Comorbidity	Drive systemic inflammation; promote progression to atopic march via gut–immune interactions.	Wang et al. [50], Mills et al. [85]	Succinate: LC-MS/MS (Liquid Chromatography–Mass Spectrometry), GC-MS mtDNA: qPCR, ddPCR (Droplet Digital PCR)
DPP-4	Serum-Specific, Endotype-Specific, Predictive	Linked to specific AD phenotypes; predicts response to IL-13-targeted therapies like tralokinumab.	Maintz et al. [10], Wollenberg et al. [87]	ELISA, Activity Assay (Fluorometric/Colorimetric)
IL-31	Serum-Specific, Severity	The “itch cytokine”, associated with pruritus but shows inconsistent correlation with disease severity.	Ozceker et al. [67]	ELISA, Luminex
Periostin	Serum-Specific, Endotype-Specific, Severity	Reflects Th2-driven inflammation; associated with eosinophilia and moderate AD severity.	Kou et al. [66]	ELISA, Western Blot
TSLP	Skin-Specific, Prognostic, Severity	Strongly correlates with SCORAD and TEWL scores; predicts barrier dysfunction.	Kim et al. [62], Nygaard et al. [63]	ELISA, Luminex, qPCR
hBD-2	Skin-Specific, Diagnostic	Elevated in psoriasis and minimally expressed in AD, aiding in differential diagnosis.	Jansen et al. [31]	ELISA, Western Blot
CXCL2, CXCL9	Serum-Specific, Predictive	CXCL9 predicts the response to cyclosporine; CXCL2 indicates the efficacy of dupilumab in high Th17 profiles.	Glickman et al. [76]	ELISA, Luminex, qPCR
IDO1	Serum-Specific, Prognostic, Comorbidity	Indicates risk of eczema herpeticum; associated with antiviral immune responses.	Staudacher et al. [82]	qPCR (for mRNA), ELISA, LC-MS/MS (for metabolites such as tryptophan and kynurenine)
CA II	Skin-Specific, Severity	Associated with skin barrier dysfunction and inflammation.	Kamsteeg et al. [24,25]	ELISA, qPCR
miR 155	Serum-Specific, Severity	Elevated levels correlate with AD severity in children.	Sonkoly et al. [45]	qPCR, Microarray
miR 203	Serum-Specific, Diagnostic	Potential non-invasive biomarker for monitoring inflammation and disease progression.	Chen et al. [46]	qPCR, Microarray
miR 483-5p	Serum/Urine-Specific, Diagnostic	Promising biomarker for non-invasive disease monitoring.	Gilad et al. [47]	qPCR, Microarray
CCL26	Serum-Specific, Severity	Linked to eosinophilic inflammation and AD severity.	Thijs et al. [55]	ELISA, Luminex
IL-17	Serum-Specific, Severity	Th17 cytokine, elevated in severe AD cases.	Yang et al. [64]	ELISA, Luminex, qPCR
KRT5	Skin-Specific, Diagnostic	Upregulated in AD; indicates keratinocyte dysfunction.	Thijs et al. [55]	qPCR, Western Blot
KRT14		Marker of basal keratinocytes; upregulated in AD lesions.		
KRT16		Overexpressed in hyperproliferative epidermis; associated with AD severity.		

## 4. Discussion

**Figure 2:**
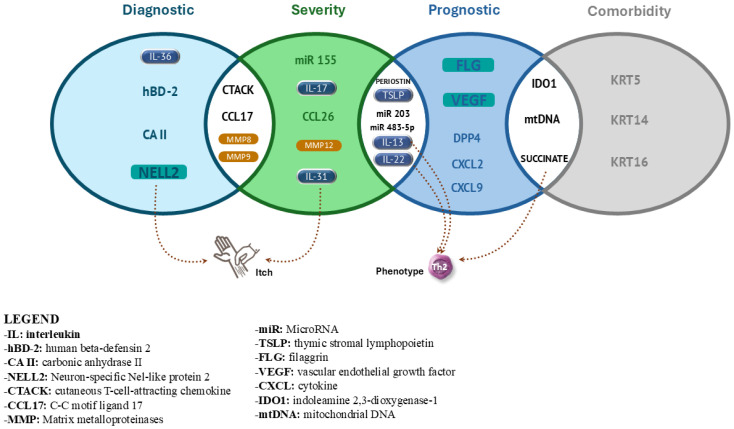
Overview of the key biomarkers in AD categorized by their role.

AD is the most common chronic relapsing disease in childhood, significantly affecting the quality of life of young patients and their families, making it a major public health concern. This issue is further compounded by the fact that AD is often one of the earliest manifestations in atopic children and, in moderate-to-severe cases with risk factors such as a family history of atopy, it is frequently associated with the development of food allergies, asthma, and allergic rhinitis [7]. Early prevention, management, and treatment of AD are therefore crucial not only for addressing the disease itself but also for mitigating the onset and progression of other atopic conditions [88,89]. The categorization of biomarkers based on their diagnostic-, prognostic-, severity-, predictive-, and comorbidity-related roles is essential for understanding their clinical relevance in atopic dermatitis. These distinctions are visually summarized in Figure 2, which provides an overview of the key biomarkers and their functional classification. In this context, using biomarkers for preventive, diagnostic, and prognostic purposes could play a pivotal role. Biomarkers help to understand disease mechanisms, enable early diagnosis, predict progression, and support personalized treatment approaches. Fifteen to twenty years ago, the identification of traditional biomarkers for AD through transcriptomics and proteomics methods marked a significant advancement in understanding the disease. During this period, transcriptomic techniques began to shed light on the gene expression profiles associated with AD (e.g., the *filaggrin* gene), revealing key inflammatory pathways and immune responses. Similarly, proteomics played a crucial role in identifying altered protein expressions, such as cytokines, chemokines, and skin barrier-related proteins. These early studies laid the groundwork for the identification of the discussed targeted therapeutic approaches [65].

Ideal biomarkers should be condition-specific, sensitive to early changes, minimally invasive (a critical aspect for pediatric populations), reproducible across diverse groups, and strongly correlated with the disease severity and outcomes. Blood samples and skin biopsies are the most commonly used biomarker collection and analysis methods, as they offer extensive diagnostic insights [90]. However, pediatric care has a clear preference for non-invasive or minimally invasive approaches to minimize discomfort and alleviate physical and emotional stress. Several non-invasive and minimally invasive methods have been developed. The innovative RNA monitoring technique offers a non-invasive approach to diagnosing and monitoring early-onset AD. This method involves analyzing RNA extracted from sebum collected through oil-blotting films. Tested on a cohort of 98 infants aged 1–2 months, the technique uncovered distinct gene expression patterns in infants with AD. These included a decrease in the genes associated with the lipid metabolism and skin barrier function, alongside an increase in immune response genes (Th2, Th17, and Th22). By detecting molecular changes that were previously inaccessible due to the invasiveness of conventional methods, RNA monitoring represents a groundbreaking, easy-to-use, and objective diagnostic tool. It enables the early detection of AD and facilitates ongoing treatment monitoring in a child-friendly, non-invasive manner [91]. Skin tape strips are used to collect superficial proteins and lipids from the skin of newborns, specifically from lesional areas. These proteins and lipids adhere to the tape, enabling non-invasive sampling for further analysis [92]. A microneedle platform is a minimally invasive tool designed to collect biological samples or deliver treatments through the skin. It uses tiny, painless needles to penetrate the superficial layers, offering a convenient and effective method for sampling or therapy while minimizing discomfort, making it particularly suitable for pediatric and sensitive populations [93]. Skin curettage is a technique used to scrape the superficial layers of the skin to collect biological material. It is a less invasive technique than skin biopsy that only scrapes the superficial layers of the skin. While a biopsy can cause pain, bleeding, and a longer recovery period, curettage is generally less painful, requires minimal recovery time, and carries a lower risk of complications [94]. Saliva is a non-invasive biological fluid which contains a variety of biomarkers, including proteins, enzymes, and genetic material. Although saliva appears to be an ideal source for biomarker measurement, there are several variables related to sample collection and handling that can affect the accuracy and reliability of the results [95,96]. According to our findings and literature research, TARC/CCL17, a Th2-type chemokine, has emerged as one of the most reliable biomarkers for AD severity, especially in pediatric populations. Its elevated levels in children strongly correlate with disease activity and progression, offering a robust measure for both diagnosis and therapeutic monitoring. Similarly, MDC/CCL22 and CTACK/CCL27 levels are significantly higher in children with AD, with correlations to disease severity, making them valuable tools for assessing inflammatory states [21,22]. Moreover, the microbiome plays a crucial role in AD, with disruptions in the skin and gut microbiota contributing to disease onset and progression. Emerging evidence highlights the link between the gut–skin axis, where gut microbiota imbalances can influence skin inflammation and barrier dysfunction [97]. Restoring and maintaining skin microbiome eubiosis has shown potential in preventing AD and reducing its severity by enhancing the skin barrier integrity and modulating immune responses [98]. Therapeutic approaches targeting microbiome balance, such as probiotics, prebiotics, and topical treatments, are gaining attention for their ability to promote long-term skin health and reduce AD flares. This emphasizes the importance of microbiome-centered strategies in managing and preventing AD. While there are not yet recommendations in this regard, studies show promising results when combined with traditional topical or injectable therapies [99,100,101]. The presence of specific microbial imbalances influences the production of biomarkers associated with AD. For example, altered microbiome profiles can affect the expression of inflammatory cytokines such as IL-22, IL-31, and TNF-α. Additionally, biomarkers related to skin barrier dysfunction, such as FLG and periostin, are impacted by microbiome changes [2,102]. Biomarkers are essential for identifying the disease endotype, allowing for the customization of therapy tailored to the patient’s specific needs. This allows healthcare providers to move away from a one-size-fits-all treatment approach and instead personalize therapy based on the patient’s unique genetic, molecular, and immune profiles. By identifying the biomarkers linked to disease progression, inflammation, or immune responses, clinicians can select more targeted and effective interventions, potentially improving treatment outcomes and reducing side effects [103].

## 5. Conclusions

Relying on a single biomarker for a complex disease like AD presents challenges. The future lies in adopting biomarker panels, which will replace traditional outcome measures and enhance the comparability of clinical trials, a crucial advancement with the growing use of biological therapies in AD treatment. In conclusion, the results underscore the transformative potential of biomarkers in understanding AD pathogenesis, improving the diagnostic accuracy, and tailoring therapeutic interventions. The multifaceted roles of these biomarkers, spanning prognostic-, predictive-, diagnostic-, and comorbidity-associated functions, offer a comprehensive approach to managing AD. Nevertheless, the integration of these findings into clinical practice demands further large-scale validation and standardization. Future research should focus on translating these discoveries into accessible and reliable tools for clinicians, ultimately enhancing patient care and outcomes in AD.
